# Patient-oriented education and medication management intervention for people with decompensated cirrhosis: study protocol for a randomized controlled trial

**DOI:** 10.1186/s13063-017-2075-4

**Published:** 2017-07-20

**Authors:** Kelly L. Hayward, Jennifer H. Martin, W. Neil Cottrell, Antara Karmakar, Leigh U. Horsfall, Preya J. Patel, David D. Smith, Katharine M. Irvine, Elizabeth E. Powell, Patricia C. Valery

**Affiliations:** 1 School of Medicine, The University of Queensland, Brisbane, QLD Australia; 2 Pharmacy Department, Princess Alexandra Hospital, Brisbane, QLD Australia; 3 School of Medicine and Public Health, The University of Newcastle, Newcastle, NSW Australia; 4 School of Pharmacy, The University of Queensland, Brisbane, QLD Australia; 5 The Centre for Liver Disease Research, Translational Research Institute, The University of Queensland, Brisbane, QLD Australia; 6 Department of Gastroenterology and Hepatology, Princess Alexandra Hospital, Brisbane, QLD Australia; 7 Statistics Unit, QIMR Berghofer Medical Research Institute, Brisbane, QLD Australia; 8 Cancer and Chronic Disease Research Group, Level 4, Central, QIMR Berghofer Medical Research Institute, 300 Herston Rd, Brisbane, QLD 4006 Australia

**Keywords:** Liver cirrhosis, Polypharmacy, Clinical pharmacist, Intervention study, Patient education, Medication reconciliation, Medication errors, Patient adherence, Medication adherence, Quality of life

## Abstract

**Background:**

People with decompensated cirrhosis require complex medical care and are often prescribed an intricate and frequently changing medication and lifestyle regimen. However, many patients mismanage their medications or have poor comprehension of their disease and self-management tasks. This can lead to harm, hospitalization, and death.

**Methods/design:**

A patient-oriented education and medication management intervention has been developed for implementation at a tertiary hospital hepatology outpatient center in Queensland, Australia. Consenting patients with decompensated cirrhosis will be randomly allocated to education intervention or usual care treatment arms when they attend routine follow-up appointments. In the usual care arm, participants will be reviewed by their hepatologist according to the current model of care in the hepatology clinic. In the intervention arm, participants will be reviewed by a clinical pharmacist to receive the education and medication management intervention at baseline in addition to review by their hepatologist. Intervention participants will also receive three further educational contacts from the clinical pharmacist within the following 6-month period, in addition to routine hepatologist review that is scheduled within this time frame. All participants will be surveyed at baseline and follow-up (approximately 6 months post-enrollment). Validated questionnaire tools will be used to determine participant adherence, medication beliefs, illness perceptions, and quality of life. Patients’ knowledge of dietary and lifestyle modifications, their current medications, and other clinical data will be obtained from the survey, patient interview, and medical records. Patient outcome data will be collected at 52 weeks.

**Discussion:**

The intervention described within this protocol is ready to adapt and implement in hepatology ambulatory care centers globally. Investigation of potentially modifiable variables that may impact medication management, in addition to the effect of a clinical pharmacist-driven education and medication management intervention on modifying these variables, will provide valuable information for future management of these patients.

**Trial registration:**

Australian and New Zealand Clinical Trial Registry identifier: ACTRN12616000780459. Registered on 15 June 2016.

**Electronic supplementary material:**

The online version of this article (doi:10.1186/s13063-017-2075-4) contains supplementary material, which is available to authorized users.

## Background

The morbidity and health care costs associated with decompensated cirrhosis are substantial because patients require complex medical care to manage debilitating complications of their disease [1, 2]. People with decompensated cirrhosis are often prescribed multiple medications for therapeutic or prophylactic use to reduce the negative health effects of cirrhosis. However, many patients lack the knowledge and skills required to contribute effectively to disease management [3, 4].

On average, this patient group has approximately three hospital admissions each year [1, 5], reflecting the high burden of illness and use of health care resources. The number of medications prescribed on hospital discharge has been found to predict rehospitalization rate and time to first hospital readmission, independently of serum biomarkers that also predict poor outcomes [2]. With recurrent admissions, more medicines are prescribed and/or the medication regimen is altered. This increases opportunities for miscommunication and nonadherence, thereby increasing the risk of medication errors and rehospitalization.

Poor knowledge of cirrhosis, medications, self-monitoring, and important dietary and lifestyle modifications has been described in ambulatory Australian patients with cirrhosis [4]. Low medication adherence was identified in over one-fourth of patients (27.5%), and discrepancies among conventional medications were also seen in 54.0% of patients with cirrhosis attending general hepatology clinics [6]. Communication barriers between patients and clinicians are a recognized impediment to patient care that may result in clinically significant discrepancies and other medication-related problems (MRPs) [7]. MRPs are events or circumstances involving medications that actually or potentially interfere with an optimum outcome of care [8]. They are complex and multifaceted issues that may be the result of numerous interacting factors. Examples of MRPs that may occur in patients with decompensated cirrhosis are presented in Table 1. It has been estimated that up to 22% of 30-day readmissions among patients with decompensated cirrhosis may be preventable with improved medication management or more frequent monitoring [2].

**Table 1:** Potential medication-related problems in patients with decompensated cirrhosis

Classification and definition	Subtype	Example
Adverse drug reaction		
A medical problem resulting from an adverse effect of a drug. These include sensitivities, intolerances, and immune-mediated hypersensitivity reactions.	Minor	Minor dizziness related to propranolol; manage with lifestyle counseling.
	Moderate	Gynecomastia related to spironolactone; may require dose adjustment or cessation.
	Severe	Stevens-Johnson syndrome precipitated by sulfamethoxazole-trimethoprim (Bactrim DS®).
Drug interactions		
An actual or potential medical problem that is related to a drug-drug or drug-patient interaction.	Drug-drug	Harvoni® (Gilead Sciences, Foster City, CA, USA) and amiodarone.
	Drug-patient	Hepatorenal syndrome precipitated by NSAID use.
Drug use without indication		
The patient is taking a medication for no medically valid reason.		Proton pump inhibitor use in a patient without gastroesophageal reflux disease, peptic ulcer, or variceal bleeding.
Incorrect dosage		
A medical condition that is being treated with drug therapy; however, the dose may be too low or too high.	Subtherapeutic	10-ml daily dose of lactulose, achieving one bowel motion every second day.
	Supratherapeutic	Spironolactone 400 mg daily in a patient with minimal abdominal ascites.
Nonadherence		
The patient is prescribed a drug for a medical condition but is not taking it for psychological, sociological, or economic reasons.	Unintentional	Forgetting to take propranolol at nighttime.
	Intentional	Not taking lactulose because of side effects (flatulence, bloating) or cost following removal from the Pharmaceutical Benefits Scheme.
Untreated indications		
A medical condition that requires drug therapy but is not being treated with medication. This may be related to intentional or unintentional nonadherence by the patient, or to intentional or unintentional underprescribing by a medical practitioner.	Nonadherence	As above.
	Underprescribing	Low cholecalciferol and tocopherol identified by pathology, not supplemented. Unintentional – oversight. Intentional – patient unable to afford currently; to be reconsidered at next visit.

*NSAID* Nonsteroidal anti-inflammatory drug

In existing models of collaborative outpatient practice, integrated pharmacist education and medication management interventions have been shown to improve patient knowledge, adherence, and outcomes in chronic diseases that have complex medication management issues similar to those of cirrhosis [9–13]. Identification of medication discrepancies and MRPs using a clinical pharmacist’s reconciled medication record reduces harm and hospitalization and may allow for simplification of the prescribed regimen [14, 15]. The association between patient knowledge, polypharmacy, poor adherence, and medication discrepancies has not been investigated in patients with decompensated cirrhosis to date. It is hypothesized that addressing this complicated relationship by implementing a targeted patient education and medication management intervention in a multidisciplinary model of hepatology ambulatory care may improve medication management, reduce MRPs, and improve medication-related outcomes for people with decompensated disease.

## Methods/design

### Study aims and hypotheses

The primary aim of the study is to determine whether the intervention (medication review, reconciliation, and additional education by a clinical pharmacist) reduces the frequency (counts) and severity (measure of clinical significance) of discrepancies between patient-reported and medical record-documented “current” medications. The secondary aims are to describe the impact of the intervention on medication adherence, quality of life (QoL), medication and illness beliefs, patient knowledge of disease and self-management tasks related to decompensation history and prescribed therapy, and the frequency of hospitalization for management of cirrhosis-related complications, non-cirrhosis-related complications, and survival. The sustainability of the intervention in the current health care system in relation to costs and cost-effectiveness will also be evaluated.

#### Hypotheses

Compared with patients receiving usual care, patients who receive the tailored (additional) education and medication management intervention are hypothesized to benefit in the following ways:

(H1) Fewer medication discrepancies

(H2) Improved medication adherence

(H3) Improved QoL

(H4) Improved illness perceptions

(H5) Improved medication beliefs

(H6) Improved knowledge of disease self-management tasks related to prescribed medications

(H7) Fewer hospital admissions

(H8) Longer survival time

### Design and setting

The study has a randomized, controlled, parallel-group design and will be conducted at a single site, the Hepatology Outpatient Centre at the Princess Alexandra Hospital (PAH), located in Brisbane, Australia. The Gastroenterology and Hepatology Department at the PAH is one of the largest hepatology centers in Australia and the only liver transplant center in Queensland. The hospital’s local catchment area is 3,856 km^2^. Additionally, many patients travel from regional and remote areas, including interstate to access specialist services. Consequently, the Hepatology Outpatient Centre is responsible for the ambulatory care of a significant proportion of southeastern Queensland patients with chronic liver disease (CLD).

### Participants

#### Eligibility criteria

Eligible participants are (1) adults aged ≥18 years; (2) diagnosed with hepatic cirrhosis by a hepatologist; and (3) current or recent (within the preceding 2 years) chronic liver failure-related complication(s), including ascites, variceal bleeding, spontaneous bacterial peritonitis, sepsis, encephalopathy, or liver-related renal dysfunction. Liver biopsy, ultrasound, and FibroScan® technology (Echosens, Paris, France) will be used to confirm the diagnosis of cirrhosis if available [16, 17]. Patient medical records and correspondence letters will be used to identify history of chronic liver failure-related complications.

#### Exclusion criteria

Individuals will be excluded for (1) inability to provide informed consent and/or (2) receiving intensive management by other health care teams (i.e., liver transplant team, palliative care).

### Participant recruitment

The Hepatology Outpatient Centre’s appointment database will be prescreened for eligible participants who are scheduled for routine review by a hepatologist. Persons identified as eligible will be contacted via telephone in the week preceding their appointment to discuss the study and offer participation. Participants will be given instructions to arrive 15 minutes early for their appointment, bring all medications to the clinic, and ask their carer or responsible family member to accompany them if appropriate. Consented participants will be randomly allocated to one of the two study arms: (1) intervention or (2) usual care.

### Randomization and allocation

An independent person at the PAH will prepare individual envelopes labeled with the randomization number and containing the corresponding study arm. Allocation concealment will be achieved by use of sequentially numbered, sealed, opaque envelopes that will be stored and opened in order by a third party located within PAH.

### Study navigation

**Fig. 1:**
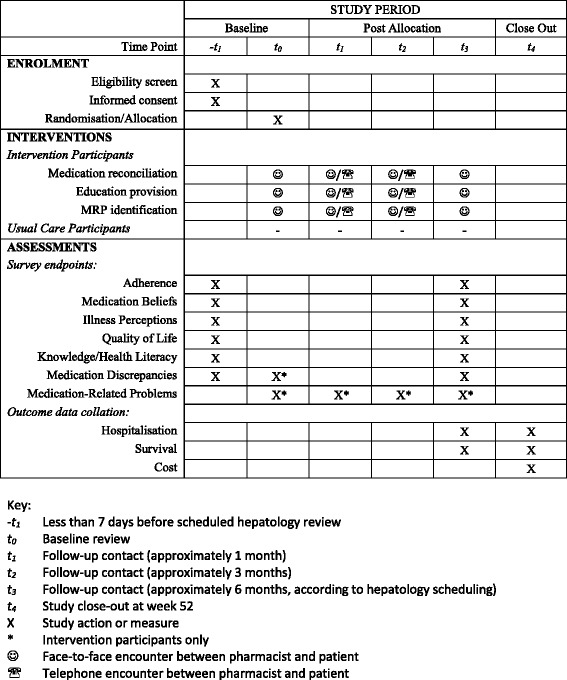
Standard Protocol Items: Recommendations for Interventional Trials (SPIRIT) flow diagram of participant recruitment and study navigation. *MRP* Medication-related problem

Figure 1 describes the planned study flow, including data collection time points. A Standard Protocol Items: Recommendations for Interventional Trials (SPIRIT) checklist of standard protocol items [18] addressed within the study protocol is provided in Additional file 1.

All participants will complete a baseline survey prior to their scheduled outpatient clinic review. Validated questionnaire tools will be used to determine baseline medication adherence, medication beliefs, illness perceptions, and QoL, in addition to patient knowledge of important dietary and lifestyle modifications. The survey will be completed with a study coordinator over the telephone or in person in a private clinic room, or it will be self-completed by the participant in the clinic waiting room. Patient carers or family members will be involved if necessary.

Usual care participants will subsequently receive routine review and education by their hepatologist according to the current model of care in the hepatology clinic. Intervention participants will be reviewed by a clinical pharmacist to receive the education and medication management intervention (described below) in addition to routine review and education by their hepatologist. Intervention participants will also receive three further educational contacts from the clinical pharmacist in addition to scheduled routine hepatologist review within the following 6-month period.

All participants will be resurveyed at follow-up (*t*_3_, approximately 6 months post-enrollment) to explore changes in their medication adherence, medication beliefs, illness perceptions, QoL, and knowledge. Patient outcome data will be collected at 52 weeks (*see* “Study endpoints and data collection” section below).

### Intervention development

A multifaceted, clinical pharmacist-driven education and medication management intervention targeted to patients with decompensated cirrhosis was developed by a panel of clinicians (including a hepatologist, general physician/pharmacologist, clinical nurse, and clinical pharmacist). Education and monitoring points (Table 2) were agreed upon on the basis of clinical hepatology guidelines to augment and support information provided by hepatologists and other clinicians involved in patient care.

**Table 2:** Medication-related education provided to intervention participants receiving pharmacotherapy for chronic liver failure-related complications

Clinical decompensation history	Advice	Monitoring
Ascites		
	Adherence with diuretics Do not add salt to food or when cooking Restaurant meals, take-out foods, and tinned foods generally contain high levels of sodium Sea salt, Himalayan rock salt, and table salt have similar sodium content Caution with salt substitutes—may contain high levels of potassium Avoid nonsteroidal anti-inflammatory analgesics Highly educated and/or engaged participants: restrict sodium intake to 2000 mg daily	Weigh self weekly if dose of diuretics is stable and ascites is well-controlled Weigh self daily if diuretic dose changes or poorly controlled ascites; notify clinic if postural symptoms/dizziness develop Document weight – do not just “remember” Do not restrict fluid intake unless advised to by a doctor Development of fevers and/or chills accompanied by abdominal pain and/or discomfort—present to the emergency department for assessment
Encephalopathy		
	Adherence with lactulose (with or without rifaximin) Do not drink alcohol Avoid sleeping tablets and opiate analgesics Do not drive unless your doctor has told you it is okay to do so Medication assistance may be required in severe memory disturbance Highly educated and/or engaged participants: discuss accumulation of ammonia and other toxins	Be mindful of worsening mood disturbances, personality changes, and sleep inversion Family member or carer to help monitor for signs of deterioration Titrate lactulose dose to achieve two or three loose bowel motions daily; reduce/withhold if diarrhea or severe bloating occurs Violent, irrational behavior or loss of consciousness—call an ambulance immediately
Hepatocellular carcinoma		Attend follow-up ultrasound appointments as scheduled
Jaundice, itch		
	Antihistamines are not very effective Use soap-free body wash Moisturize skin daily	Get blood tests as directed Acute or recurrent yellowing of eyes and skin—see a doctor as soon as possible
Malnourishment		
	Eat small, frequent meals regardless of appetite Eat a bedtime snack Choose protein- and calorie-rich snacks Take supplements as directed Use an antiemetic if required Do not drink alcohol	Weigh self once weekly Keep a food diary See a dietitian for expert advice
Variceal bleeding		
	Adherence with propranolol (with or without proton pump inhibitor) Highly educated and/or engaged participants: discuss portal hypertension and development of varices	Attend endoscopic surveillance as scheduled Monitor blood pressure, postural symptoms, dizziness Check hemoglobin and iron stores as appropriate Hematemesis or melena—call an ambulance immediately

### Intervention description

During the initial encounter with each intervention patient (and carer/family member if present), the clinical pharmacist will obtain a complete, reconciled list of current medications and identify MRPs. The pharmacist will use open and closed questions to ascertain adherence, other medication-taking behaviors, and medication needs on an individualized basis. Participating patients will receive medication- and lifestyle-related education and advice tailored to their needs (decompensation history and medications) and receptiveness (Table 2). The pharmacist will also collaborate with the patient’s general practitioner and hepatologist to optimize therapy, facilitate resolution of MRPs, and monitor issues where required.

Subsequent contact with participants will be made at 4–6 weeks (*t*_1_) and 12–14 weeks (*t*_2_) after the baseline interview to discuss changes in prescribed therapy; identify new MRPs and previously identified MRPs that are ongoing; reiterate advice provided at baseline; and provide additional relevant education about medications, lifestyle modifications, and self-management of cirrhosis to build on previous discussions. Promotion of a patient-clinician partnership will be central to each discussion to encourage functional health literacy and self-efficacy with regard to patient confidence to raise questions and discuss issues with health care providers.

### Medication-related problems

During patient interviews, the clinical pharmacist will identify actual and potential MRPs and document these in the patients’ medical notes and on a structured data collection form. The pharmacist will then facilitate the resolution or monitoring of individual MRPs with other relevant clinicians as appropriate and document each outcome in patients’ medical notes and data collection forms.

**Fig. 2:**
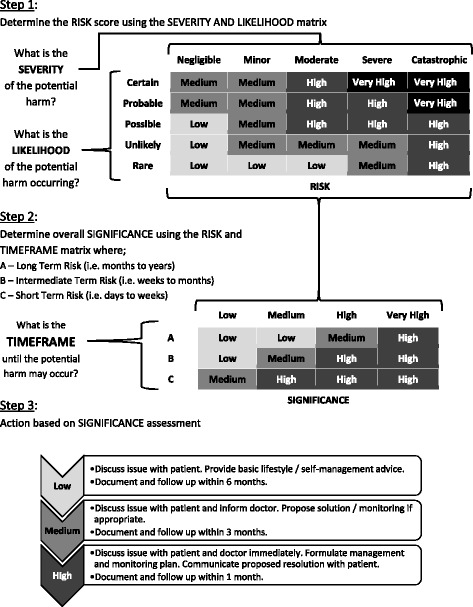
Risk matrix for assessment of significance of medication discrepancies and other medication-related problems

The clinical significance of MRPs (and medication discrepancies) will be assessed by a clinician panel using a risk matrix (Fig. 2) modified from the Metro North Health Service Risk Management Framework [19]. The matrix assigns a composite risk of prospective severity and likelihood of potential harm, as well as duration of time until potential harm may occur, as a consequence of the MRP. The panel of clinicians will comprise at least one clinical pharmacist, hepatologist, and general physician/pharmacologist who have experience in managing patients with cirrhosis. MRPs will be de-identified, randomized, and independently assessed by at least two members of the panel. Relevant clinical information will accompany the de-identified MRPs, but not details of patient outcomes or MRP resolution, to ensure unbiased prospective assessment of potential harm. Consensus of individual rankings will be used to determine the final measure of potential harm. Where there is disagreement between individual rankings, a roundtable panel discussion will be called to facilitate consensus.

### Tailored intensity intervention

Intervention participants will receive education and advice tailored to their needs, receptiveness, and cognitive barriers. For example, a patient who has nonalcoholic steatohepatitis with well-controlled abdominal ascites and no other decompensation history would likely require a low-intensity intervention if he or she identified as adherent. Low-intensity intervention for this patient would consist of reiteration of important dietary modifications, monitoring of weight, and diuretic education. Conversely, a malnourished ex-alcoholic patient with a history of poorly controlled ascites, recurrent encephalopathy, and persistent hypotension will require high-intensity intervention. This would ideally involve inclusion of a carer/family member in the discussion to educate both parties on the potential precipitants and early signs of encephalopathy, self-titration of lactulose, and when to present to the emergency department. Pharmacotherapy education (including possible discussion of adherence aids), high-protein and low-sodium dietary modifications, daily weight monitoring (including when to contact the clinic if weight is changing significantly over short periods of time), and review of pharmacotherapy that may contribute to hypotension would also be conducted. Liaison with additional health providers may also be required.

After providing education and advice to intervention participants, the clinical pharmacist will use a structured data collection form to record topics that have been discussed, topics that were not discussed and reasons why, and whether a carer was present. Post hoc testing will be employed to explore study outcomes according to the intensity of the intervention received and the completeness of relevant information provided (*see* the “Data analysis” section below).

### Medication reconciliation

Using information from several sources (including the patient, general practitioner, pharmacy, own medications, carer), the clinical pharmacist will construct a reconciled list of current pharmacotherapy. The reconciled list of medications will be made available to other clinicians in the patient’s medical record, and a patient-friendly guide will be constructed using the approved statewide template. This guide will be printed and provided to the patient. The patient will be encouraged to take it to all health care appointments and use it at home to aid with compliance and memory. The guide will also contain additional medication management and lifestyle points related to prescribed therapy, such as low-salt diet reminders for patients who are prescribed diuretics in the management of ascites. Patients will be encouraged to manually edit the medication guide when therapy is altered.

### Study endpoints and data collection

Study outcomes, tools, and time points for measurement are outlined in Table 3. Follow-up will occur approximately 6 months post-enrollment (allowed range 140–224 days after baseline interview), according to outpatient hepatology scheduling availability. If a follow-up appointment is missed, a second appointment will be offered to the patient within the allowed time frame. If the second appointment is missed, the patient will be considered lost to follow-up for *t*_3_ survey endpoints.

**Table 3:** Outcomes and other measures of the medication management and education intervention

Categories/variables	Measure	Collection week
Medication discrepancies		
Between drug name and dosage Frequency of per-patient discrepancies Severity of discrepancies	Patient interview Questionnaire Medical record review	*t*_0,_ *t*_3_
Medication-related problems (intervention arm only)		
Adverse drug reaction Drug interaction Untreated, undertreated, or overtreated indication Other medication-related barriers	Patient interview Questionnaire	*t*_0,_ *t*_3_
Adherence		
Self-reported medication adherence Affordability	MMAS-8 Patient interview	*t*_0,_ *t*_3_
Quality of life		
Health-related quality of life Abdominal and systemic symptoms Emotional functioning, fatigue, and worry Daily activity	CLDQ Brief IPQ	*t*_0,_ *t*_3_
Beliefs and perceptions		
General medication beliefs Beliefs about liver disease medications Illness perceptions	BMQ-General BMQ-Specific Brief IPQ	*t*_0,_ *t*_3_
Knowledge/health literacy		
Knowledge of causation Dietary modification including sodium restriction Self-monitoring requirements Safe analgesia Source of medication-related information	Questionnaire Brief IPQ	*t*_0,_ *t*_3_
Hospitalization		
Liver-related hospitalization Non-liver-related hospitalization Duration of stay	Medical record review	*t*_3,_ *t*_4_ (and 3 years)
Survival		
Liver-related mortality Non-liver-related mortality	Medical record review	*t*_3,_ *t*_4_ (and 3 years)
Cost		
Cost of service implementation Potential cost savings	Cost modeling	After study completion

*Abbreviations: BMQ* Beliefs about Medicines Questionnaire, *Brief IPQ* Brief Illness Perception Questionnaire, *CLDQ* Chronic Liver Disease Questionnaire, *MMAS-8* Eight-question Morisky Medication Adherence Scale

### Primary outcome

#### Medication discrepancies

Discrepancies between patient-reported and clinician-documented current medications will be obtained via the patient questionnaire (medication recall), patient interview (reconciliation for intervention participants only), and medical record review (clinician documentation). The clinical significance of discrepancies will be determined by blinded comparison at the completion of the study using a risk matrix tool (Fig. 2), which assigns a composite ranking of severity, likelihood, and duration, as described in the Methods subsection “Medication-related problems” above. Discrepancies (between medications, doses, and frequency of administration) will be examined at baseline and follow-up (*t*_3_) for both groups. A composite endpoint of frequency (counts of) and potential risk will be examined by measuring total frequency of high/very high risk discrepancy occurrence and the frequency of high/very high risk discrepancy occurrence per patient.

### Secondary outcomes

A questionnaire will be used to collect data on the secondary outcomes of interest at baseline and follow-up (*t*_3_). Validated tools used to measure these endpoints will be included in the questionnaires with approval from their respective developers.

#### Adherence

Change in self-reported medication adherence will be examined using the eight-question Morisky Medication Adherence Scale (MMAS-8) [20–22] and patient interview. The MMAS-8 has been validated extensively in other chronic diseases and multiple languages worldwide [21–23]. It contains seven questions with yes/no alternatives and one question that features a 5-point Likert scale. Adherence scores can range from 0 to 8 and may be trichotomized into high (score 8), medium (score 6 to <8), and low (score <6) adherence rankings.

#### Quality of life

The Chronic Liver Disease Questionnaire (CLDQ) [24] is a widely used health-related QoL tool that has been validated in numerous etiologies and severities of CLD and in several languages [24–29]. The CLDQ asks patients to rank a series of symptoms, impacts on daily activities, and mood disturbances for frequency of occurrence using a 7-point Likert scale, where a higher score indicates a better QoL. It includes 29 items across 6 domains: 8 questions related to emotional function; 3 questions related to abdominal symptoms; 3 questions related to activity; and 5 questions within each of the domains of systemic symptoms, fatigue, and worry.

#### Medication beliefs

Beliefs about medications will be examined using the Beliefs about Medicines Questionnaires (BMQ-General and BMQ-Specific) [30], which contain a series of statements about medications. Patients are required to indicate the extent of their personal agreement or disagreement with each statement using a 5-point Likert scale. The BMQ-General contains four items related to the overuse of medicines and four items about medication harmfulness. The BMQ-Specific contains five items about “necessity” beliefs and five items about “concerns” that patients may have about their medicines. The internal consistency, discriminant validity, and correlation of the BMQ with other clinical measures are described elsewhere [30–32].

#### Illness perceptions

The Brief Illness Perception Questionnaire [33] will be used to examine changes in patients’ perceptions about cirrhosis. The questionnaire has proven validity and correlation with outcomes in numerous chronic diseases [33, 34]. It contains eight items that patients rank from 0 to 10 related to their perception of disease and medicines. Patients are also asked to identify three important factors that they believe caused their current illness.

#### Cirrhosis and lifestyle knowledge

Knowledge of cirrhosis self-management tasks related to decompensation history and prescribed medications will be determined via questionnaire (using the same questions pre- and postintervention). Eight questions related to over-the-counter analgesia, management of ascites and sodium restriction, and monitoring of weight and blood pressure have been incorporated into the surveys, adapted from Volk et al. [3].

#### Hospitalization and mortality

The number of hospital admissions, including the frequency of abdominal paracenteses in patients with ascites, will be derived from medical records. Hospitalizations for liver-related and non-liver-related events in the 2 years preceding recruitment and 12 months following recruitment, including data on patients’ clinical decompensation prior to enrollment and postenrollment (e.g., date, type and severity of decompensation event, new or recurrent event) will be collected. Follow-up will be conducted at 12 months and again at 3 years. Frequency and duration of hospitalization for management of cirrhosis-related and non-cirrhosis-related complications, cirrhosis-related mortality, and non-cirrhosis-related mortality will be interpreted within the context of patients’ liver disease severity, decompensation history, development of new decompensation events, and other prognostic and clinical variables.

#### Medication-related problems

Intervention participants may have MRPs identified during their interview that warrant follow-up action or monitoring. The pharmacist will document the types of MRPs identified, the action or monitoring required, and the steps taken toward resolution in patients’ medical records and using the structured data collection tool. This information will not be available for usual care participants, because they will not have a medication review. The change in frequency (count) and potential harm (risk matrix) of MRPs will be assessed using an equivalent time sample where the intervention group is its own control. The clinical significance of MRPs will be determined by blinded comparison at the completion of the study using a risk matrix tool (Fig. 2), as described in the Methods subsection “Medication-related problems” above.

#### Cost-effectiveness analyses

Economic evaluation of the study intervention (with respect to potentially prevented hospital admissions, harms, reduced mortality, quality-adjusted life-years gained, and so forth) will be performed in collaboration with health economic analysts at the conclusion of the study.

### Safety measures for serious adverse events

Study progress, collected data, and clinical cases will be reviewed periodically by the study investigators. Frequency and causation of participant hospitalization and mortality will be reviewed at 6, 12, and 18 months because the study group of interest is at high risk for these occurrences. If significant differences are found between the intervention and control groups, or if serious adverse events develop throughout the study, an independent data safety board will be convened to review the appropriateness of continuing the study in accordance with the Australian National Health and Medical Research Council National Statement on Ethical Conduct in Human Research [35].

### Intervention personnel

#### Clinical pharmacist

The intervention will be conducted by a tertiary hospital-trained clinical pharmacist who has experience working in a hepatology unit and has received education from a hepatologist regarding symptoms, signs, and clinical significance of liver decompensation events. Ongoing continuous education relevant to hepatology and the management of liver patients will be undertaken throughout the study period and will consist of weekly meetings with hepatology consultants and advanced trainees as well as attendance at departmental case discussions, journal club meetings, and in-service education.

#### Senior clinician supervision

The intervention will be facilitated by specialist medical and nursing staff to provide clinical guidance and supervision to the intervention pharmacist. The pharmacist will work in collaboration with the senior hepatologists and the hepatology clinical nurse coordinator to identify and resolve MRPs and optimize therapy. At least one supervising hepatologist will be present in the clinic during the pharmacist’s working hours. Recommendations to modify therapy will not be communicated to the patient without prior discussion with a supervising clinician. The study protocol and patient education provided to participants have been approved by the clinical leadership.

### Data analysis

Data will be coded and stored in a password-protected database and analyzed using a statistical software package (IBM SPSS Statistics version 20.0; IBM, Armonk, NY, USA). Study endpoints experienced for each treatment group will be formally analyzed on an intention-to-treat basis [36]. Important potential confounders will be included in a secondary multivariate analysis (e.g., hospital admission, mortality). Post hoc subgroup analysis will be used to further explore outcomes according to the intensity of the intervention and completeness of relevant personalized education received by intervention participants.

The study endpoints will be presented quantitatively (proportions, statistical differences between groups) and qualitatively (description of discrepancies, MRPs, and some patient-reported medication beliefs and illness perceptions). All tests will be two-tailed, and significance will be set at α = 0.05. Continuous and normally-distributed variables will be presented as mean ± SD. Differences between groups will be analyzed using one-way analysis of variance. Non-normally distributed data will be presented as median (range), and nonparametric tests will be used to analyze differences between groups. Categorical data will be analyzed using Pearson’s chi-square test or Fisher’s exact test. Pearson’s correlation or Spearman’s rank correlation coefficient will be used to identify linear relationships between normally distributed and non-normally distributed continuous variables, respectively.

Differences in the study endpoints of interest will be examined between the intervention and control groups at baseline and at follow-up. The endpoints will be examined at baseline to test for homogeneity between groups (effectiveness of randomization) and to identify clinical variables that may influence endpoints (e.g., Model for End-Stage Liver Disease score; Child-Turcotte-Pugh classification; decompensation history, including event date, type, severity, and recurrence; hospitalization frequency; age; sex; biochemical abnormalities; and other prognostic variables). Clinically significant differences at baseline between groups will be adjusted for during the final analysis. Changes in the frequency of medication discrepancies, self-reported adherence, QoL, medication beliefs, illness perceptions, and patient knowledge at follow-up will be examined to measure the effect of the intervention on study outcomes at group and individual levels. The primary endpoint of medication discrepancies will be explored using total frequency of instances and frequency of instances per patient and per medication group. Comparison between intervention and control groups at baseline and *t*_3_ will be made using total number of instances per group, total number of high/very high risk instances per group, mean/median number of instances per patient, and mean/median number of high/very high risk instances per patient.

Predictors of clinical outcomes will be identified using binary logistic regression to determine the OR (95% CI). A multivariable logistic regression model will be used to determine independent predictors for patient outcomes. The multivariable logistic regression model will be constructed using variables of clinical significance (severity of disease; decompensation history, including date, type, severity, and frequency of events; other prognostic variables) and statistical significance as determined by univariate analysis.

Time to first episode of decompensation and time to death (cause-specific and all-cause) will be assessed using Kaplan-Meier survival curves. The curves will be compared with the log-rank test statistic. Cox proportional hazards modeling will be used to calculate HRs, after testing the proportional hazards assumption. Patients will contribute person-time from the date of recruitment until the earliest of end of follow-up (12 months) or death (date of death to be obtained from medical records). Competing risks analysis will be performed in which we encode, as separate events, time to first readmission with a cirrhosis-related episode, death resulting from cirrhosis, or death resulting from other causes. This will be extended to multivariate analysis if there are a sufficient number of events.

It is anticipated that a proportion of participants (approximately 30%) will be lost from the study before completing follow-up assessments at *t*_3_. Although the sample size calculation has been adjusted to accommodate for this loss, subjects who have completed only one assessment will be included in the analyses to establish baseline parameters in both treatment groups. With respect to changes from baseline, data imputation will be considered. In decompensated cirrhosis, dropouts due to death are informatively censored, and thus the responses of patients with missing data will be modeled explicitly. Subjects will be matched using baseline demographic and patient-reported outcomes, repeating this technique for both intervention arms. Imputation methods such as a hot-deck imputation and iterative regression imputation will be used. The sensitivity of the results to each of these methods will be assessed to quantify the degree of bias present.

### Power calculation and sample size determination

On the basis of a primary endpoint of a 50% reduction in medication discrepancies (3.12 ± 2.5 per patient [6] to 1.56 per patient), 41 participants will be required per treatment arm to achieve ≥80% statistical power. Median survival in decompensated cirrhosis is 2 years [37]. Therefore, a 6-month mortality rate between 10% and 15% among the study cohort is predicted. The Hepatology Outpatient Centre’s attendance failure/reschedule rate is approximately 20%. Therefore, a possible 30% loss will be accounted for, and at least 106 participants will be recruited.

## Discussion

Authors of meta-analyses and researchers in randomized controlled trials have found that clinical pharmacist interventions improved achievement of therapeutic targets (via improved adherence and medication titration) in people with diabetes [10], hypertension [38, 39], and hyperlipidemia [40]. In heart failure, pharmacist intervention identified and resolved MRPs [11], improved adherence [11], optimized medication titration [11, 41], and reduced all-cause mortality and heart failure-related hospitalizations [12]. Similar benefits were identified in renal and transplant groups [9, 13]. Addressing MRPs, discrepancies, nonadherence, and patient knowledge deficits related to prescribed therapy may also improve outcomes for patients with decompensated cirrhosis.

Volk et al. hypothesized that improved patients’ understanding of their medications may prevent a significant proportion of 30-day readmissions among patients with decompensated cirrhosis [2]. However, patient understanding of disease and therapy is a difficult variable to ascertain and modify because it may be affected by numerous interacting dynamic domains, including health literacy, beliefs about health and medicines, patient-clinician interaction (including quality of education and communication), self-efficacy, and the influence of other internal and external barriers [31, 32, 42]. These barriers, which may be social or economic factors, clinician or health care system factors, or patient-related factors, can further impact adherence [43]. People with poor health literacy, those living in disadvantaged areas, or those from culturally and linguistically diverse backgrounds comprise a large proportion of the CLD cohort [44–47]. These patients often have poor comprehension of chronic disease, including limited ability to access and use health information to make effective decisions about their health care without guidance [48]. Therefore, the intervention requires flexibility in its delivery and intensity to accommodate patients from a broad range of backgrounds.

Medication education, reconciliation of the medication history, and identification of MRPs are considered valuable clinical contributions by pharmacists to a patients’ care [14]. Medication discrepancies have been identified in over 50% of ambulatory patients with cirrhosis and were associated with poorer adherence and polypharmacy [6]. MRPs have not been formally investigated in decompensated liver disease, but prevalence is thought to be high. Up to 12% of medical admissions annually in Australia have been associated with MRPs [15]; however, the risk of medication errors may be further compounded in people with advanced cirrhosis by impaired alertness and memory due to low-level chronic encephalopathy. This reduced cognitive activity can negatively impact disease insight, memory, and medication adherence. The education and medication management intervention must consider these potential cognitive barriers and involve the carer or a family member where appropriate.

Low health literacy has not been a barrier to improving health knowledge and medication management behaviors, though strategies and interventions to promote self-efficacy have been more effective in improving clinical status than information provision alone [42]. Illness and medication beliefs have been shown to influence patients’ confidence to self-manage alcoholic liver disease and to have impacts on health-related QoL [49]. Progression of disease is associated with decreased QoL, including increased symptoms; abdominal symptoms and fatigue have been found to worsen adherence in people with cirrhosis [50]. Investigation of the effect of these variables on medication adherence in patients with decompensated cirrhosis, in addition to the effect of a clinical pharmacist education and medication management intervention on modifying these variables, will provide valuable information for future ambulatory management of these patients.

### Limitations

In this study, we will evaluate the effectiveness of a single pharmacist in one general hepatology clinic in Australia, and applicability of findings to other sites will be dependent on similarities in patient demographics and models of care. An important component of this study is the provision of an intervention that is tailored to the patient’s needs, receptiveness, and cognitive barriers. As a consequence of the “reactive” nature of the intervention, the information provided will be selective, and delivery will be staggered over the course of the study. Perceived variability in delivery may be due to lack of clinical indication (i.e., not providing information on precipitants of hepatic encephalopathy to patients who have never had encephalopathy), time restraints, patient disinterest, or “information overload.” Where possible, advice and education points will be reiterated and built upon during future contacts.

There will be heavy reliance on patients’ self-reported information, such as the details of their local pharmacy and general practitioner for the process of medication reconciliation; self-reported adherence; QoL; beliefs; and perceptions. This and other clinical information will also be dependent on honest patient responses to the survey questionnaires. Although this is acknowledged as a limitation, patient reporting remains the most important method of information gathering in clinical practice in terms of eliciting history and clinical status. Furthermore, self-reported adherence using the MMAS-8 has been shown to be equally as accurate as and less onerous than other methods of adherence measurement, including pill counts. Where necessary, supplemental information will be sought from patients’ family members or other carers to corroborate patients’ responses if the study clinicians have concerns about patient safety in accordance with good clinical practice.

### Trial status

At the time of proof review, recruitment into the study is complete.

## Additional file

Additional file 1: SPIRIT 2013 checklist [18]: recommended items to address in a clinical trial protocol and related documents. Page numbers identified within the checklist correspond to the location of specified Standard Protocol Items within the original manuscript. (DOC 120 kb)

## Abbreviations

BMQ: Beliefs about Medicines Questionnaire
Brief IPQ: Brief Illness Perception Questionnaire
CLD: Chronic liver disease
CLDQ: Chronic Liver Disease Questionnaire
MMAS-8: Eight-question Morisky Medication Adherence Scale
MRP: Medication-related problem
NSAID: Nonsteroidal anti-inflammatory drug
PAH: Princess Alexandra Hospital
QoL: Quality of life
SPIRIT: Standard Protocol Items: Recommendations for Interventional Trials
